# Shared processing in multiple object tracking and visual working memory in the absence of response order and task order confounds

**DOI:** 10.1371/journal.pone.0175736

**Published:** 2017-04-14

**Authors:** Mark D. Lapierre, Simon J. Cropper, Piers D. L. Howe

**Affiliations:** Melbourne School of Psychological Sciences, The University of Melbourne, Parkville, Victoria, Australia; Monash University, AUSTRALIA

## Abstract

To understand how the visual system represents multiple moving objects and how those representations contribute to tracking, it is essential that we understand how the processes of attention and working memory interact. In the work described here we present an investigation of that interaction via a series of tracking and working memory dual-task experiments. Previously, it has been argued that tracking is resistant to disruption by a concurrent working memory task and that any apparent disruption is in fact due to observers making a response to the working memory task, rather than due to competition for shared resources. Contrary to this, in our experiments we find that when task order and response order confounds are avoided, all participants show a similar decrease in both tracking and working memory performance. However, if task and response order confounds are not adequately controlled for we find substantial individual differences, which could explain the previous conflicting reports on this topic. Our results provide clear evidence that tracking and working memory tasks share processing resources.

## Introduction

Despite living in a dynamic environment, with retinal images that are continuously changing, observers can create and maintain a coherent perceptual representation of their external environment. To create this representation, observers need to not only identify and label individual objects but also to keep track of their whereabouts. Even without eye and head movements this is a significant task. The work described in this paper is focused on the interaction between the two processes required for this task: tracking moving objects and remembering their identities. For observers to know which objects are located where in the visual environment, these two processes must interact coherently and effectively. How this happens remains a contentious issue. Here, we review several recent studies and present our own to show how their seemingly disparate conclusions can be brought together to form a more cogent view of the shared processing in tracking and working memory.

### Working memory

Working memory may be broadly defined as the temporary storage and manipulation of mental representations. It enables individuals to perform complex tasks such as reading and interacting with the environment. Working memory processes include, at the very least, encoding, maintaining, and retrieving representations of stimuli or abstract mental constructs [1, 2].

Many apparently simple visual tasks involve keeping track of more than one moving stimulus in the scene; the ongoing perceptual coherence of the world requires this process to occur constantly [3]. Previous research has supported a significant role of working memory in object-tracking, evidenced by the interference between tracking tasks and a variety of working memory tasks both behaviourally [4–6], and neurophysiologically through electroencephalography (EEG) [7]. Although each of these studies has concluded both tasks draw upon a common resource, as one might intuitively expect, the degree and parameters of that interaction remain unclear. The details of the interaction are essential to understanding how the visual system represents multiple moving objects, and how those representations contribute to tracking and to working memory tasks [8–11].

### Multiple-Object Tracking (MOT)

**Fig 1:**
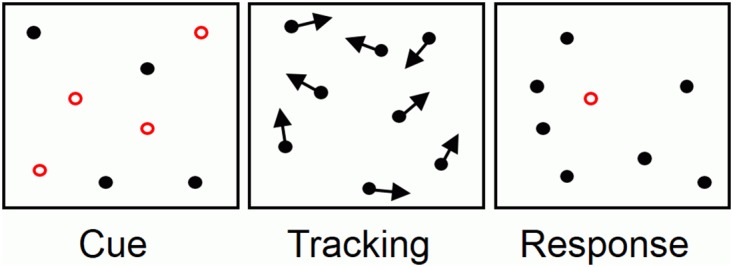
An example of a typical Multiple Object Tracking (MOT) task. The targets to be tracked are indicated during the cue phase, e.g., by flashing red for 1.5 seconds. The cues disappear and all objects begin moving independently during the tracking phase for a limited time, e.g., 6 seconds. Finally, during the response phase, the observer is prompted to indicate the location of the targets. In this example one object is probed and a ‘yes’ or ‘no’ response would be made. The square boundaries are shown only for illustrative purposes; the stimuli are usually bounded by the screen dimensions.

Typically, tracking performance is evaluated using the Multiple Object Tracking (MOT) task, which requires sustained, distributed attention directed towards simple, dynamic, highly controlled, visually identical stimuli [12]; illustrated in Fig 1. MOT tasks have mostly been used to study attention [13], but they have also provided evidence of a role for visual short-term memory in tracking. Studies have shown that tracking improves when objects are visually unique [14–16], and degrades when performed concurrently with a task that requires visual short-term memory [4, 5, 14]. One model of tracking argues that long-term memory also contributes to tracking by facilitating the process of binding each target to its current location [17].

### Working-memory and tracking

Several studies have paired an MOT task with various secondary tasks selected for their distinct working memory demands, with the aim of discerning which aspects of working memory are involved in MOT [4–6, 18, 19]. There is general agreement from these studies that tracking tasks and memory tasks do compete for similar resources [4–6]. Conceptually, any processes that demand attention will compete for common resources, and several studies have shown that the distribution of attention across tasks depends upon factors such as the precise task, its demands upon spatial and temporal processing [5, 11, 18], and the particular way in which the dual-task design is implemented [6]. The degree of interference between attention-demanding processes therefore depends on the exact way attention is distributed between the two tasks [6]. A recent study, using EEG recording, implied a dual process involved in MOT, with only one of the processes (the ‘indexing’ mechanism) competing with Visual Working Memory (VWM) for attentional resources [7]. Thus, only some aspects of the two tasks might interfere with each other [7]. It appears that the nature of the interference between the two tasks is complex and strongly stimulus and task-dependent. The work described here aims to disentangle some of these factors and provide a clearer picture of the degree to which MOT and VWM compete for the same attentional resources.

### The current study

We take as a starting point the observation by Zhang and colleagues [6] that previous findings of disruption to MOT performance could be accounted for by the requirement to make a response to the VWM task prior to the MOT task response [4, 5, 18]. They suggested that the requirement to make a response may be more disruptive than the task itself. However, it is unclear what it is about making a response that is disruptive. The current study aimed to clarify this point and extended the previous work in two important ways.

Firstly, unlike the work cited above, the difficulty of the MOT task was calibrated in both experiments for each participant so that the task would be sufficiently challenging to compete with the concurrent VWM task for any shared resources. This necessitated a single-subject design for the experiments rather than the group-design more commonly adopted in the literature.

Second, there were two response conditions; one in which the VWM response was requested before the MOT response [5], and another in which the MOT response was requested before the VWM response [6]. If MOT and working memory share processes beyond those required for making a response to a task, then evidence of interference should not be solely attributable to response-order effects. Specifically, if making a response is the dominant source of disruption, MOT performance would be expected to be equivalent in single- and dual-task conditions when the MOT response is made first, but dual-task MOT performance will be worse than single-task performance when the VWM response is made first. If competition for shared resources also contributes to disruption, but only when resources are adequately consumed by both tasks, dual-task performance for both MOT and VWM tasks should be worse than single-task performance for each respective task, regardless of the order in which the responses are made.

Three experiments are reported here. Experiments 1a and 1b form a clear empirical link to the work discussed above. In particular, VWM performance in previous research was well below ceiling [4–6], so similar stimulus parameters were adopted for Experiment 1a for parity with these studies. Conversely, in Experiment 1b, both MOT and VWM tasks were calibrated for each participant to allow a better assessment of the interaction between the two tasks. For Experiment 2, we refined the stimulus to allow us to better estimate dual-task costs. In this way we are able to sequentially examine the critical stimulus parameters in the interaction between the two tasks and relate our conclusions directly to previous work.

## General methods

### Participants

There were six participants (B.S., U.B., S.L., A.M., P.C., and R.M.; three female) in Experiment 1a, four (A.S., B.S., U.B., and A.L.; one female) in Experiment 1b, and five (M.L., A.S., S.S., Z.T., and R.Y.; one female) in Experiment 2. B.S. and U.B. participated in Experiments 1a and 1b, while A.S participated in Experiments 1b and 2. All were reimbursed for their time and provided informed written consent (with the exception of the author, M.L.). The study was approved by the Department Human Ethics Advisory Group in the Melbourne School of Psychological Sciences at the University of Melbourne. Each experiment of this study was conducted over multiple (around 10) sessions, with each session lasting between 45 minutes and one hour.

### Apparatus

The participants viewed the stimuli on a 21-inch CRT monitor (Sony G520) at a resolution of 1600 by 1200 pixels with a frame rate of 100 Hz at a distance of 68 cm. Stimuli were presented in MATLAB [20] using the Psychophysics Toolbox [21].

### Stimuli and procedure

Experiments 1a and 1b involved similar stimuli; only the response phase of the paradigm was different between the two. Experiment 2 involved a few substantial changes to the stimuli, which are described in a subsequent section.

**Fig 2:**
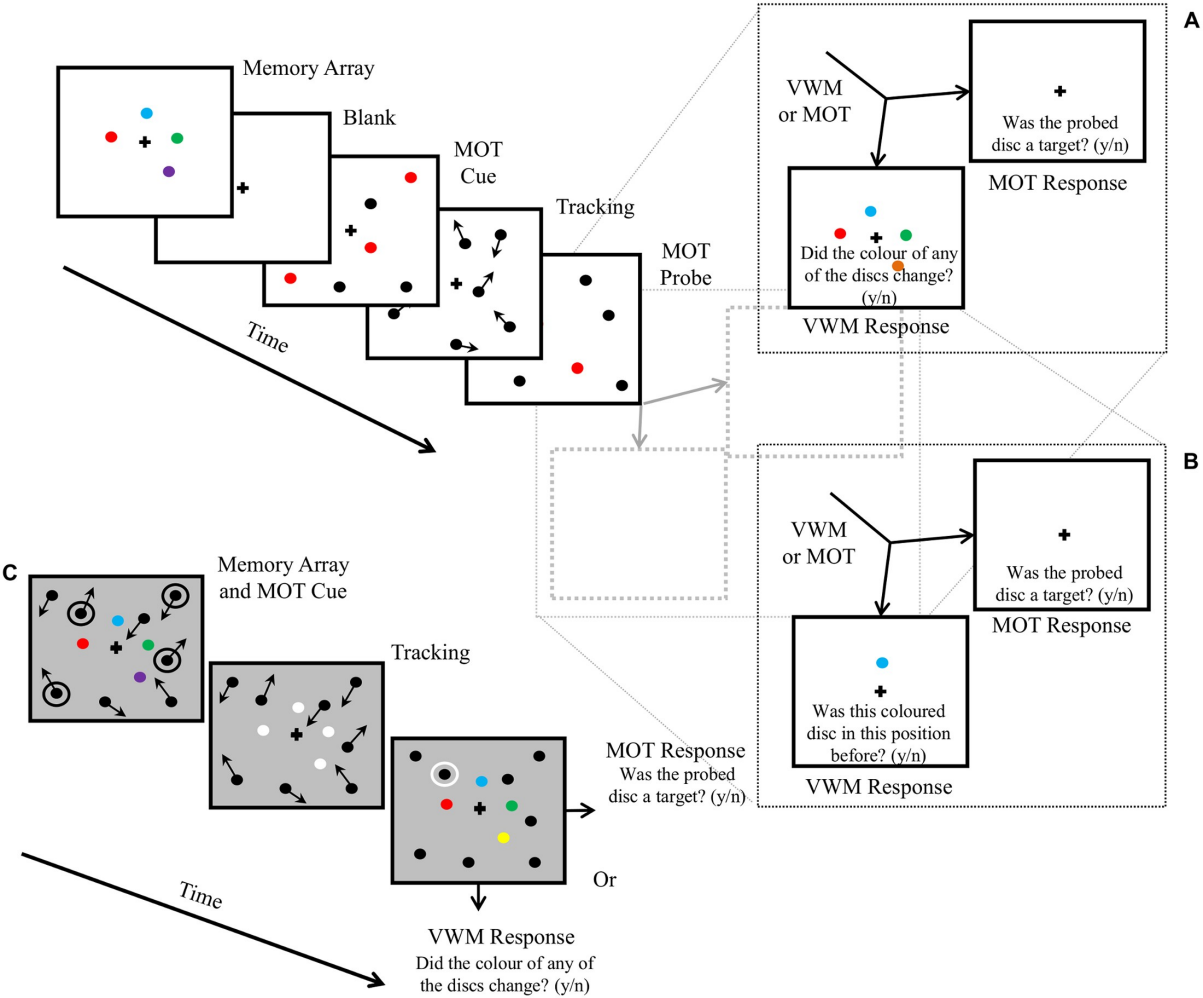
A diagram of the stimuli used in all Experiments. A) In Experiment 1a the memory array of the visual working memory (VWM) task was displayed first, followed by a brief blank period before the multiple object tracking (MOT) task began. The MOT cue phase began with the discs stationary for 1 second, and then moving for 2 seconds. The tracking phase proceeded for 5 seconds, followed by the MOT probe phase, and finally the response phase. On 50% of trials the MOT response would be requested, and on the other 50% of trials the VWM response would be requested. B) In Experiment 1b the full array of stimuli was displayed during the VWM response phase, not the single probe item that was displayed in Experiment 1a. C) In Experiment 2 both the MOT and VWM components of the display were presented concurrently throughout the trial.

In Experiments 1a and 1b the stimuli were composed of twelve discs of one degree of visual angle (°) in diameter displayed on a white background. Eight of the discs were the objects of the MOT task and the remaining four discs comprised the memory array of the VWM task. These two disc sets were displayed at different times and did not overlap (see Fig 2). The region in which the discs could be displayed was an imaginary rectangle that subtended 31° by 24°. A black fixation cross was displayed in the centre of the screen, subtending 0.5° by 0.5°. The CIE co-ordinates of the point of the background were x = 0.271, y = 0.284. The luminance of the white background and black fixation cross were, respectively, 33.3 cd/m^2^, and 0.2 cd/m^2^.

Observers were required to perform either the MOT task or the VWM task alone, or both tasks simultaneously. Regardless of whether tasks were performed under single-task or dual-task conditions the stimuli were the same prior to the response phase. When performing the MOT task alone the VWM stimuli were still displayed and when performing the VWM task alone the MOT stimuli were still displayed. In single-task conditions the observers were instructed to ignore one set of stimuli and were only asked for the relevant response. The stimuli are represented in Fig 2 and described below.

#### VWM test phase

At the start of each trial the four discs of the VWM task were displayed at four randomly chosen locations from among seven equidistant possible locations on an imaginary circle with a radius of 5°, centred on the fixation cross. They were each coloured red, green, blue or purple, each disc a different colour (see Fig 2), the observers’ task was to remember the array, knowing they would be asked about the colour of one item at the end of the stimulus interval. The discs disappeared after 0.5 seconds and a blank screen was then displayed for 1.2 seconds.

#### MOT test phase

The eight discs of the MOT task then appeared at randomly assigned positions, at least 2.5° from any other disc. Four of the discs were cued as the targets to be tracked by being displayed red. The remaining four distractor discs appeared black. Although the red was the same red as used in the VWM array, pilot testing reassured us that there was no conflict created by the use of this colour between the two tasks when presented at different times, as was the case in Experiments 1a and 1b. Experiment 2 used a different colour as stimuli were coincident. The discs were stationary for one second after which the tracking phase began, with discs moving in random directions at a constant speed. After another two seconds of movement the red target discs became black, identical to the distractors. The discs were not permitted to collide; whenever a disc came within 2.5° of the centre of another disc both discs appeared to bounce off an invisible buffer surrounding each disc. Discs also bounced off the walls of the imaginary rectangle. The discs continued to move for six seconds before stopping, at which point one of the discs was probed for one second by being coloured red. The observer was required to make a judgment as to whether the probed disc was a target, but they were not required to respond yet.

#### Response phase

There were four options for the response phase of Experiments 1a and 1b: MOT only, VWM only, MOT first, and VWM first. During the response phase, if the observer was performing the MOT task alone only the MOT response would be requested, but if the observer was performing the VWM task alone only the VWM response would be requested. If the observer was performing both tasks then both responses would be requested. In the *MOT-response-first* condition the MOT response would be requested first, followed by the VWM response. The order was reversed in the *VWM-response-first* condition. In all conditions, chance performance was 50%. The response phase of Experiment 2 involved a different arrangement of stimuli and is described in a later section.

In the MOT response phase the following question was displayed, “Was the probed disc a target (y/n)?” The observer would press the ‘y’ key if they had judged the probed disc to be a target, otherwise they would press the ‘n’ key (chance performance 50%).

In Experiment 1a, during the VWM response phase one of the discs comprising the VWM stimuli appeared again and the following question was displayed, “Was this coloured disc in this position before (y/n)?” On 50% of trials the disc would appear at the location at which one of the other discs had previously appeared, for the other 50% of trials the disc would appear at the original location.

In Experiment 1b the VWM probe phase revealed the entire memory array rather than a single disc. On 50% of the trials one of the discs changed colour to a colour that was not included in the original memory array. This change was made so that the VWM probe phase revealed all objects involved in the task, similar to the MOT probe phase, which also revealed all objects involved in the task. The probe array was displayed for one second immediately after the MOT probe, rather than after the MOT response if the MOT response was made first. This manipulation avoided the variable VWM maintenance periods that the unspeeded MOT response generated in Experiment 1a. It also equated the probe durations of the MOT and VWM responses, although the task durations still differed. The VWM response prompted the observer to answer, “Did the colour of any of the discs change (y/n)?” The appropriate response was made via key press for Experiments 1a and 1b, as per the MOT response phase.

#### Task calibration—MOT calibration (all experiments)

Prior to performing the task proper, each observer underwent a calibration phase to determine the speed at which the MOT task discs should move such that a correct response could be made on approximately 75% of trials. A two-stage process was used in which performance data was collected using the method of constant stimuli, and the QUEST algorithm [22] was used to estimate psychometric function slope and threshold. During the first stage the observer saw discs moving at five speeds randomly and uniformly distributed over 50 trials, with speeds chosen to cover a wide range from chance to perfect performance, based upon the observer’s report of previous experience with MOT-like tasks. This ranged from a minimum of 2°/s to a maximum of 20°/s. The data from this stage were entered into the QUEST algorithm to estimate the psychometric function slope and to roughly estimate the 75% performance threshold. The observer then entered the second stage, performing another 50 trials at five speeds of a narrower range centred on the estimated 75% threshold. Finally, all 100 trials from both stages were plotted, outliers removed, and the remaining data submitted to QUEST to estimate a 70% threshold (lower than 75% to account for further improvement expected during the experiment).

#### Task calibration—VWM calibration (Experiments 1b and 2)

Participants underwent an additional calibration phase for the VWM portion of the tasks in Experiments 1b and 2. Similar to calibration for the MOT portion of the tasks, the aim was to determine the stimuli parameters that would allow the observer to accurately respond to approximately 75% of trials. This was achieved by varying the number of discs the observer was required to memorise. The observer saw three to seven coloured discs, with the number of discs randomly and uniformly distributed over 50 trials. The discs were displayed at randomly chosen points from among a set of equidistant possible locations on an imaginary circle with a radius of 5°, centred on the fixation cross. The number of possible locations was always three more than the number of discs, for example, if a trial displayed five discs, the locations would be chosen from eight possible locations. The data from this stage were input into the QUEST algorithm to roughly estimate the 75% performance threshold. The observer then entered the second stage, performing another 50 trials at three values for the number of discs to be memorised, with the value centred on the roughly estimated 75% threshold. Finally, all 100 trials from both stages were plotted, outliers removed, and the remaining data were submitted to the QUEST algorithm to estimate a 70% threshold (lower than 75% to account for further improvement expected during the experiment). Calibration was performed under single-task conditions. MOT calibration was performed first followed by VWM calibration with MOT objects moving at the speed determined during MOT calibration.

### Data analysis

In Experiments 1a and 1b analyses were conducted in accordance with Fisher’s procedure [23]. Specifically, a one-way repeated measures ANOVA was conducted to test for an effect condition (single-task, dual-task MOT-response first, dual-task VWM-response first), separately for MOT performance and for VWM performance. If the test was significant all pairwise comparisons (*t*-tests) were performed at the alpha level of the omnibus test (.05). If the ANOVA was not significant, it was assumed there was no difference between conditions and planned comparisons were not conducted. Levin and colleagues [23] demonstrate that in the specific case of comparisons between three conditions, Fisher’s procedure ensures that the family-wise error cannot exceed the prescribed alpha level. Experiment 2 involved a factorial design and therefore Fisher’s procedure is inappropriate. Bonferroni adjusted significance levels are reported.

#### A note on study design

When considering the results below, it important to bear in mind that this study is fundamentally a single-subject design and although we will report the group data as well as individual data, we can only be sure of our conclusions when all subjects agree with the group overall, at least in trend. This is a departure from the norm; the previous studies cited reported the group analysis, but each individual—even though there were more of them—did far fewer trials. We consider this a critical part of the problem we are addressing here and we examine the relationship between the individuals and the group mean as part of the analysis.

## Results and discussion

### Experiment 1a: Calibrated MOT task

**Fig 3:**
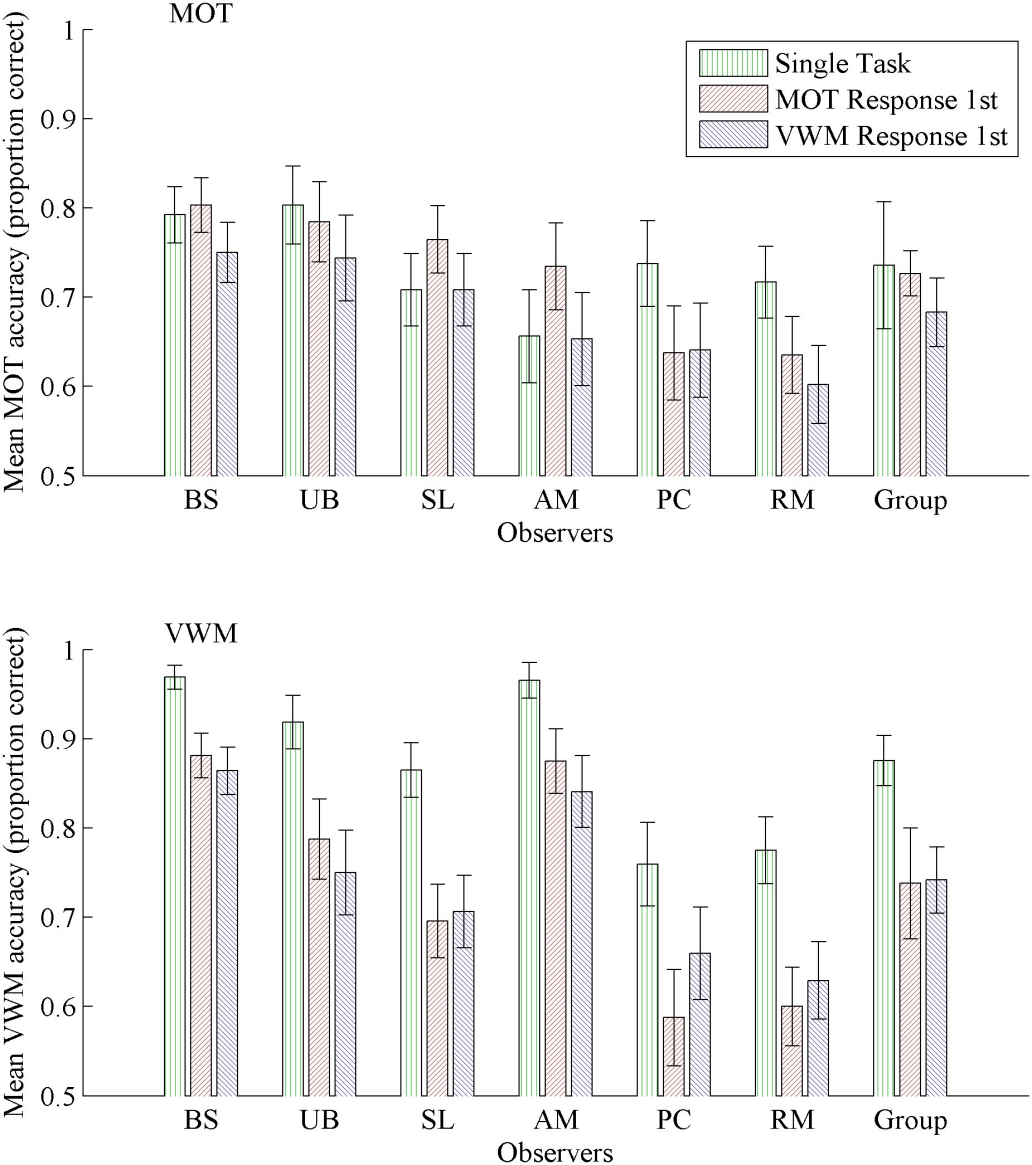
Experiment 1a: Individual and group performance. Mean accuracy in each condition for the multiple object tracking (MOT) task shown in the top panel, and for the visual working memory (VWM) task in the bottom panel. Group analysis, to the far right in each panel, is consistent with previous research, however individual performance shows substantial variation, particularly in the MOT task. Error bars represent 95% confidence intervals.

Fig 3 plots the performance of each participant in the MOT task (top panel) and the VWM task (bottom panel) for each of the response conditions; Tables 1 and 2 give the individual and group numerical data. A repeated measures ANOVA of all observers’ data revealed a marginally significant effect of condition on MOT performance, *F*(2, 10) = 3.48, *p* = .07. Tracking was better in the single-task condition than in the VWM-response first condition, but not the MOT-response first condition. Tracking in the MOT-response first condition was better than in the VWM-response first condition. Individual observers’ data deviated substantially from this general pattern (see Table 1), with only B.S. conforming for all comparisons. Observer U.B. was similar, but performance in the MOT-response first and VWM-response first conditions did not differ significantly, which it did for observer B.S. Analyses for observers S.L. and A.M. indicated superior performance in the MOT-response first condition than in either the single-task or VWM-response first conditions, but no different between the latter two conditions. Observers P.C. and R.M. showed more accurate tracking in the single-task condition than in the MOT-response first or VWM-response first conditions, but no difference between the latter two conditions.

**Table 1:** Repeated measures ANOVA comparing of MOT performance between conditions for each observer.

Observer	Degrees of freedom	Single vs. MOT first		Single vs. VWM first		MOT first vs. VWM first	
		*t*	*p*	*t*	*p*	*t*	*p*
B.S.	1278	0.49	.63	1.80	.07	2.28	.02
U.B.	638	0.59	.56	1.80	.07	1.21	.23
S.L.	958	1.98	.048	0	1	1.98	.048
A.M.	638	2.15	.032	0.08	.93	2.23	.026
P.C.	638	2.74	.006	2.66	.008	0.08	.93
R.M.	958	2.70	.007	3.77	.002	1.06	.29
Group	5	0.31	.077	2.73	.04	3.79	.01

**Table 2:** Repeated measures ANOVA comparing of VWM performance between conditions for each observer.

Observer	Degrees of freedom	Single vs. MOT first		Single vs. VWM first		MOT first vs. VWM first	
		*t*	*p*	*t*	*p*	*t*	*p*
B.S.	1278	6.02	<.001	6.89	<.001	0.92	.35
U.B.	638	4.77	<.001	5.89	<.001	1.12	.26
S.L.	958	6.44	<.001	6.08	<.001	0.35	.72
A.M.	638	4.29	<.001	5.46	<.001	1.24	.21
P.C.	638	4.71	<.001	4.71	<.001	2.80	.005
R.M.	958	5.95	<.001	5.00	<.001	0.93	.35
Group	5	8.27	<.001	11.54	<.001	0.22	.84

A repeated measures ANOVA revealed a significant effect of condition on VWM performance, *F*(2,10) = 51.96, *p* < .001 (Fig 3 bottom panel). Change detection performance was greater in the single task condition than in either the MOT-response first or VWM-response first conditions. There was no difference between MOT-response first and VWM-response first conditions. Individual observers’ data was qualitatively similar to the group analysis (see Table 2), except for P.C. who showed more accurate change detection performance in the VWM-response first condition compared to the MOT-response first condition.

The pattern of results for the group analysis (right-hand side bar triplet in Fig 3) appears to support the hypothesis that making a response is the dominant source of disruption to MOT, while VWM is less resilient against disruption because the MOT task is performed during the VWM task’s maintenance phase. Dual-task MOT performance was worse than single-task MOT performance only when the VWM response was made first, not when the MOT response was first. VWM dual-task performance was disrupted to an equivalent extent regardless of which response was made first.

As flagged above, while these averaged results conform to the pattern reported previously [5, 6], individual observer analyses show substantial variation. Only two observers, B.S. and U.B., displayed the same pattern of results. Observers S.L. and A.M. showed inexplicably greater dual-task MOT performance compared with single-task MOT performance when the MOT response was made first. Finally, observers P.C. and R.M. showed worse dual-task performance than single-task performance for both tasks, and equivalent dual-task performance regardless of response order. It is also noteworthy that single-task VWM performance was close to ceiling for all but these latter two observers. This pattern of results lends support to the alternative hypothesis; that competition for shared resources also contributes to disrupted performance, but is only observable when both tasks are sufficiently demanding of those resources.

### Experiment 1b: Calibrated MOT and VWM task

Experiment 1b aimed to further investigate the alternative hypothesis by calibrating VWM task performance as well as MOT task performance such that each observer would perform at a level of approximately 75% correct under single-task conditions. As before, if competition for shared resources also contributes to disruption, but only when adequately consumed by both tasks, dual-task performance for both MOT and VWM tasks should be worse than single-task performance.

#### Results and discussion

**Fig 4:**
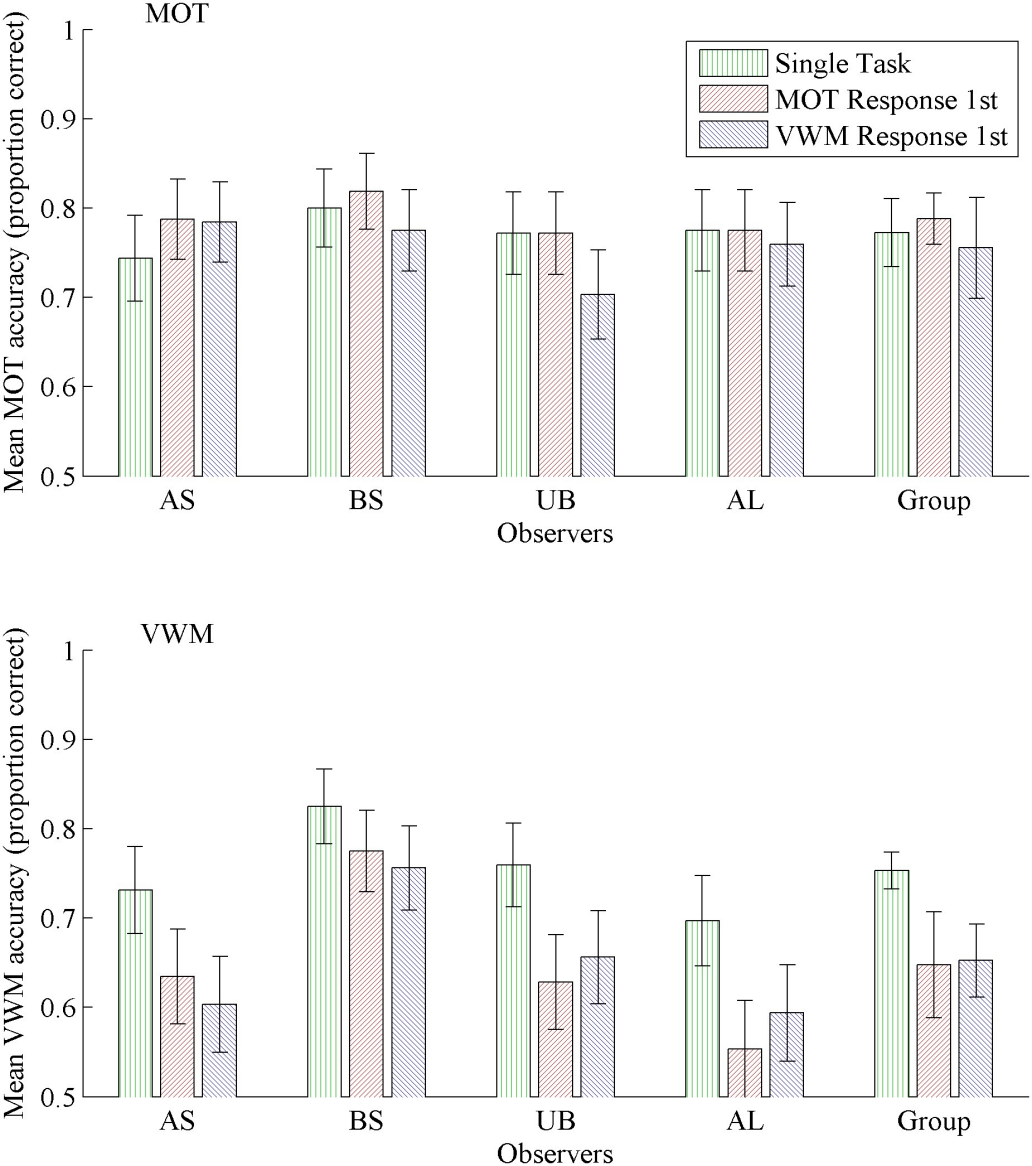
Experiment 1b: Individual and group performance when both tasks were calibrated separately for each individual. Mean accuracy in each condition for the multiple object tracking (MOT) task shown in the top panel, and for the visual working memory (VWM) task in the bottom panel. Group analysis to the far right in each panel. Error bars represent 95% confidence intervals.

Fig 4 plots the individual and group data in a format consistent with Fig 3 and Table 3 gives the numerical data. When VWM and MOT tasks were both equally demanding, a repeated measures ANOVA of all observers’ data did not reveal an effect of condition on MOT performance (Fig 4 top panel); unlike the significant, albeit only slightly, effect in Experiment 1a above. Analyses of almost all individual’s data via logistic regression similarly failed to reveal an effect of condition on MOT performance, except for observer U.B., *β* = 0.18, *t*(958) = 2, *p* = .045. For U.B., tracking accuracy was worse in the VWM-response first condition than in either the single-task condition, *t*(638) = 1.98, *p* = .048, or the MOT-response first condition, *t*(638) = 1.98, *p* = .048.

**Table 3:** Repeated measures ANOVA comparing of VWM performance between conditions for each observer.

Observer	Degrees of freedom	Single vs. MOT first		Single vs. VWM first		MOT first vs. VWM first	
		*t*	*p*	*t*	*p*	*t*	*p*
A.S.	638	2.64	.008	3.47	.006	.81	.42
B.S.	638	1.58	.11	2.14	.03	.56	.57
U.B.	638	3.63	<.001	2.88	.004	.74	.46
A.L.	638	3.79	<.001	2.74	.006	1.04	.30

NB similar analysis of MOT performance revealed no significant effects (see text).

Conversely, a repeated measures ANOVA revealed a significant effect of condition on VWM performance, *F*(2,6) = 23.75, *p =* .001 (Fig 4 bottom panel), similar to the results of Experiment 1a. Change-detection performance was greater in the single-task condition than either the MOT-response first, *t*(3) = 5.02, *p* = .02, or VWM-response first, *t*(3) = 8.27, *p* = .003, conditions. There was no difference between MOT-response first and VWM-response first conditions, *p* = .81. Individual observers’ data was qualitatively identical to the group analysis (see Table 3), except for B.S. who showed no difference between change detection performance in the single-task condition compared with the MOT-response first condition.

The results do not appear to support the prediction that simply making both tasks challenging enough to avoid ceiling effects under single-task conditions would reveal dual-task interference for both tasks. Again, the individual’s patterns of data are most revealing. Three observers, A.L., A.S., and B.S., showed no difference between conditions for MOT performance, reflecting the group analysis. Note that B.S. participated in the previous experiment, as did U.B., although she showed the same pattern of MOT performance; equivalent performance for single-task tracking accuracy and for dual-task tracking when the MOT response was made first, but poorer accuracy when the VWM response was made first compared to the single-task condition. Observer U.S. also showed the same pattern for the VWM component of the task as for her performance in the previous experiment; the MOT-response first condition and VWM-response first condition both showed less accuracy than the single-task condition. Observers A.S. and A.L showed the same pattern of VWM performance, reflecting the group analysis, while B.S. showed greater performance in the single-task condition than in the dual-task condition when the VWM response was made first, but not when the MOT response was made first. The unexpectedly high dual-task performance in this experiment and the previous may be the result of an effect of arousal, or possibly an effect of general task improvement for observer B.S. It is also possible that the order in which tasks were performed biased observers’ allocation of attention towards the MOT task and away from the VWM task; a bias that might shift with experience. Whatever the underlying reason(s), the individual differences make clear conclusions very hard to draw and again caution strongly against relying only on group data. While it would be worthwhile to resolve these unexpected results for specific individuals, the more immediate question remains that of whether MOT and VWM tasks share the same processes to a substantial extent. The results of Experiments 1a and 1b provide conflicting results, suggesting a strong role for response-order effects for some observers, but not so for others.

These individual differences mirror the differences between previous studies where the group data were presented [4–6, 18], suggesting there may be another factor, or factors, at play, which we address in Experiment 2.

### Experiment 2: Overcoming both response and stimulus confounds

A potential problem that may be contributing to these differences in results, between individuals or studies, is that of the VWM task being performed first, with the MOT task being performed wholly within the maintenance period of the VWM task, thus rendering the VWM task potentially more likely to be disrupted than the MOT task. Experiment 2 avoids these confounds by having both tasks displayed and performed simultaneously, and by asking for only one response per trial, aiming to produce a clearer picture of the interaction between processes involved in both tasks. If task order or response order are the dominant causes of interference between tasks, with those causes removed single-task performance should be equal to dual-task performance for both MOT and VWM. However, if dual-task deficits in performance are a result of the use of shared resources or processes, and observation of such deficits depends on sufficient consumption of resources in the absence of response- and task-order confounds, dual-task performance is expected to decrease for both tasks relative to single-task performance. Apparatus and participants were as in Experiment 1 except where indicated.

#### Stimuli and procedure

Two participants, M.L. and A.S., had enough prior experience with tracking tasks that maintaining a performance level of approximately 75% would have required an excessive increase in the speed of the discs. To compensate for this the number of MOT discs was increased, relative to the previous experiments, to ten for all participants; five targets and five distractors. Calibration was conducted for each participant as described in the General Methods section. The stimuli were displayed in stages similar to the MOT portion of the previous experiments, except that the cue and probe phases were combined. The MOT cues and the VWM memory array were displayed simultaneously at the start of each trial. The MOT discs were black and the targets were cued by being surrounded by a white circle (visible against the grey background). This cue was used rather than the previous red colour cue to avoid confusion between MOT targets and a red disc in the memory array, as mentioned in the General Methods section. The discs of the VWM array were uniquely coloured (red, green, blue, purple) for approximately 1.2s after which they changed to white to distinguish them from the MOT discs, and remained visible and stationary until the response phase. The exposure duration was longer compared to previous experiments to account for the increased difficulty of performing both tasks simultaneously. The MOT discs bounced off the VWM discs as well as each other. The proximity buffer of 2.5° was maintained. During the combined-probe phase the MOT discs stopped moving and one was surrounded by a white circle; a target on 50% of trials, a distractor otherwise. The VWM probe array appeared simultaneously, displaying the original memory array, except that on 50% of trials one of the discs would be a colour that did not appear at the start of the trial. During the response phase one of two questions would appear, (1) “Was the circled disc a target (y/n)?”, or (2) “Did the colour of any of the stationary discs change (y/n)?” Each question appeared on 50% of trials, randomly assigned. During single-task conditions the observers were instructed to ignore one set of stimuli and were only asked for one relevant response. This is illustrated in Fig 2C.

#### Results and discussion

**Fig 5:**
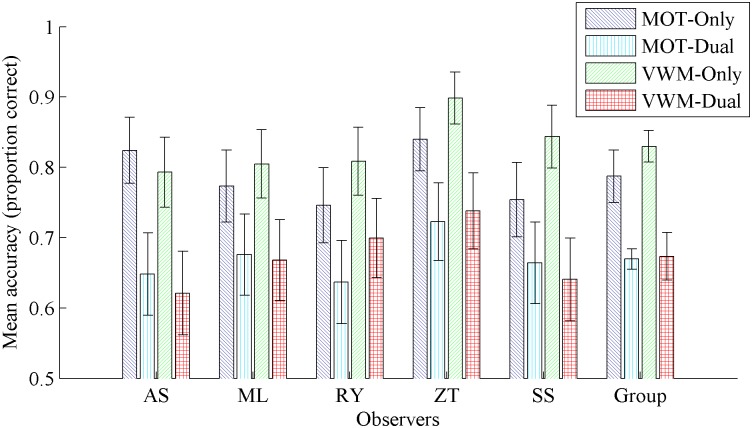
Experiment 2: Mean accuracy in each condition. Single-task and dual-task performance for each observer for the multiple object tracking (MOT) and visual working memory (VWM) tasks. Group analysis to the far right. Error bars represent 95% confidence intervals.

Results are shown in Fig 5 in a format consistent with the previous two figures and numerically in Table 4. A response (MOT, VWM) by load (single, dual) ANOVA of all observers’ data revealed a main effect of load, *F*(1, 16) = 54.49, *p* < .001. There was no main effect of response and no interaction between load and response. Accuracy was greater under single-task conditions compared to dual-task conditions for MOT performance and VWM performance. Planned comparisons were analysed via logistic regression for each individual’s data, and *t*-tests for group data. Significance was tested against a Bonferroni adjusted alpha level of .004 (.05/12). For the majority of observers, performance was qualitatively identical to the group data (See Table 4); however, the difference between single- and dual-task MOT performance did not reach the conservative Bonferroni adjusted alpha-level for observers M.L. and S.S, and was marginally significant for observer R.Y. A significant interaction was found for observer S.S., *β* = 0.67, *t*(1020) = 2.30, *p* = .02; under single task conditions VWM performance was greater than MOT performance, *β* = .57, *t*(1020) = 2.52, *p* = .01, however under dual task conditions there was no difference, *p* = .58.

**Table 4:** Comparison of single- and dual-task performance for MOT and VWM tasks for each observer.

Observer	MOT performance Single vs. Dual			VWM performance Single vs. Dual		
	*β*	*t*	*p*	*β*	*t*	*p*
A.S.	.93	4.44	<.001	.85	4.22	<.001
M.L.	.49	2.46	.014	.72	3.48	.001
R.Y.	.52	2.67	.008	.60	2.85	.004
Z.T.	.70	3.18	.001	1.14	4.55	<.001
S.S.	.44	2.23	.026	1.11	5.13	<.001
Group		9.5	<.001		12.05	<.001

*Note*: Individual analyses conducted via logistic regression with 1020 degrees of freedom for each test. Group analysis conducted via *t*-test with 5 degrees of freedom.

The stimulus configuration used here (and summarised in Fig 2, Experiment 2) maximises the effect of the task-driven influences on performance, e.g., the shared demands of the tasks, while minimising stimulus-driven effects, e.g., the speed of the movement of the MOT discs. Even when the calibration for each individual was significantly reduced in a second set of subjects there is a group trend toward mutual interaction and the same effect shown in some of the subjects (see S1 File for details). Overall, the results of Experiment 2 suggest that when the stimulus configuration, task order and individual differences in single task performance are accounted for, the differences between the subjects are largely removed and a moderate but consistent effect of the two tasks on one another is seen.

## General discussion

The overall aim of this study was to gain a greater understanding of the interaction between attention and memory processes in MOT by evaluating the extent to which the attention-demanding task of tracking is disrupted when a VWM task is performed simultaneously. Although previous research had demonstrated mutual disruption [4–7, 11], it remained possible that the order in which tasks were performed, or the simple act of making a response, were responsible for the observed disruption, rather than competition for resources required for the task *per se* [6]. The work described here sought to clarify this issue experimentally by removing stimulus-driven influences and, further, scaling the stimuli to individual differences in baseline performance in order to most clearly measure the task-driven interactions between the two processes; attention and memory.

At first blush, the results of Experiment 1a appear compatible with previous research [5, 6]; tracking was disrupted under dual-task conditions, but in general only when observers responded to the VWM task first. Consistent with previous research, VWM performance was disrupted under dual-task conditions regardless of response order. However, when individual subject performance was analysed three distinct patterns of performance were revealed, otherwise obscured by the group analysis. For example, for two individuals’ performance in both MOT and VWM tasks was disrupted under dual-task conditions regardless of response order. Experiment 1b tested the hypothesis that if both tasks were sufficiently challenging and the stimuli displayed during the probe and response phases were more closely matched, then mutual interference would be more readily observed; this was not the case. The results of Experiments 1a and 1b provided conflicting results, suggesting a strong role for response-order effects for some observers, but not for others. Although individual differences could explain some of the variance [10, 15], task-order and response-order confounds may have continued to contaminate conclusions regarding dual-task interference.

Experiment 2 was designed to avoid those confounds by having both tasks start simultaneously, by simultaneously displaying probe stimuli for both tasks, and by requesting only one response. The results revealed disruption in both tasks for each participant, confirming the hypothesis that if the influence of response order and task order were removed, interference would be observed in the performance of both tasks. Note that for some participants, dual-task MOT performance was not significantly different from single-task performance at a Bonferroni adjusted alpha level. However, given the highly conservative nature of Bonferroni correction, and the consistent pattern of results across observers, these findings nonetheless lend support to the conclusion that MOT and VWM tasks employ shared processes that are not restricted to the processing of spatial information alone [4, 5], and that attention has a central role that is shared with non-visuospatial tasks [18]. So even though it was previously found that MOT performance was not disrupted by a concurrent VWM task, except when that task involves processing of spatial-location information [6], the current findings suggest a more general conclusion. Shared processing of spatial location information is more likely to result in disruption to performance, however, a VWM task that involves non-coincident spatial processing may still disrupt a tracking task, if both tasks exhaust the resources available to them. If processing resources were truly independent, performance would not suffer as long as the task design itself did not interfere with task performance or interaction; Experiment 2 of the current study fits these criteria. Particular care was taken in the design of the combined to task to ensure the VWM and MOT elements did not interfere either in appearance (colours vs identifying rings) or location; a strict 2.5° separation was maintained throughout [24] with only the MOT elements in motion. In terms of the VWM task, our stimuli, once calibrated, were equivalent to the ‘compact’ structure used by Zhang and colleagues [6] in their Experiment 2, in that the spatial location of the VWM elements did not change between trials. From an MOT perspective, we controlled for ‘swapping’ errors between the VWM task and the MOT task by ensuring two element arrays never got too close to each other, and the MOT stimuli were calibrated for ‘dropping’ errors by making speed the independent variable in calibration [8]. Each of these factors reassures us that while the tasks and stimuli were concurrent, they did not interfere with each other through stimulus-driven factors; only through the task-driven factors of primary interest, which clearly showed a mutual interaction.

We emphasise that the calibration of task difficulty for each subject was critical to ensure that both tasks were demanding of attentional resources and equally so. So although this made the tasks individually harder than in previous research, we argue that this is the only way to adequately answer the question at hand; are the same resources shared when both tasks are performed concurrently? If either task is able to draw on resources that are not used by the other task, then it is possible that performance will appear impervious to disruption. As Experiment 1a shows, the group data suggest MOT performance was not disrupted but VWM performance was. However, single-subject data revealed that MOT performance was in fact disrupted for two individuals. This highlights one of the drawbacks of only using group data in such studies and we also argue that one of the strengths of the current study is the single-subject design. Despite this we have used between 4 and 6 subjects depending upon the experiment; generally above the current average for single-subject psychophysics (see the S1 File for data from an additional 6 subjects).

Our data show significant individual differences in performance, which has been a hallmark of the literature to date and has proven useful in the theoretical analysis of capacity limitations in working memory [25], though it is often obscured through the presentation of group data. When we examine the individual-subject results, we progressively reduce the differences in performance between subjects through our experimental progression by calibrating to individual baseline performance (1a-1b) and removing stimulus-based confounds in the experimental paradigm (1b-2). The effect of reduced calibration time shown for the second set of subjects reported in S1 File suggests that the scaling of task difficulty and, by proxy, resource demand is an essential component of this individual difference.

### Models of working memory

Within the literature cited here the two descriptive models of working memory most commonly invoked to explain the data are the multicomponent working memory model (M-WM) [1, 2, 26, 27] and the more integrated model suggested originally by Cowan [28–31]. It is a matter of ongoing debate how significantly the two approaches differ from each other [2, 32] though the explicitly defined components the M-WM lead to its greater popularity as a descriptive framework in which to couch the data. The current study, in common with all others cited here, is not able to not discriminate between these two approaches; our data is consistent with aspects of both. The result of Experiment 2, i.e., that there is consistent mutual interaction between the two tasks, is predicted by both models in their current form.

There are other popular models of memory, but they tend to focus on other aspects of the overall process and less on the working-memory stage(s) of most interest in the current context [33–35]. That is not to say that these alternative theories do not have a role in our overall understanding of the processes of memory, nor that they do not influence one another in their respective theoretical progression, as they clearly do [2]. It is also the case that more general theories of attention and integration of information must be involved to provide a full account of the underlying processes [35–38], and understanding the complex nature of working memory in all its forms remains an ongoing challenge [25]. In light of this, and for the purpose of the current discussion of what is primarily an empirical paper, we will focus on the M-WM [1, 2, 26, 27] as has the majority of the immediately relevant literature [4–7].

To briefly review the M-WM structure, its four components are the central executive, the phonological loop, the visuospatial sketchpad, and the episodic buffer. In the current study, VWM performance was readily disrupted regardless of response order, suggesting it shares cognitive resources with MOT of the kind for which the visuospatial sketchpad is responsible, and that it relies on these to a substantial degree. Once task difficulty, task order and stimulus issues were dealt with in Experiment 2, all subjects showed mutual disruption, galvanising earlier conclusions [4–7]. These data are consistent with the broad strokes of the M-WM model, but do not inform whether VWM and MOT tasks share the central executive, the episodic buffer, an additional subcomponent of the visuospatial sketchpad other than that responsible for spatial processing, or some combination of those components. The mutual interactions seen in the data could happen at any stage and are symptomatic of the repeatedly observed capacity limits on memory and attention.

Both MOT tasks and VWM tasks require significant overt attention during some part of each task itself; namely the initial phase of indexing or identification of target properties in both MOT and VWM [5, 11], during tracking when there is the potential to confuse, or ‘swap’, a target with a distractor in an MOT task [8, 39], and during maintenance in a VWM task [40]. The episodic buffer would be involved in all of these cases, as it is responsible for the storage of episodic representations that bind information from different modalities or different features of the same object [40], as would the central executive, due to the attention-demanding nature of the tasks [2].

It has been suggested that only part of the MOT task, indexing rather than tracking, demands common (memory) resources with a VWM task [7], which suggests a greater role for the episodic buffer in the M-WM. However, during the maintenance of the two tasks, while there was no explicit overt stimulus to draw attention, the act of tracking to avoid errors and making eye movements to facilitate tracking makes it unlikely that the MOT task was ever only using the memory-independent resources suggested to mediate tracking alone [7, 11]. Related to this observation, a study of distractor load in MOT and VWM suggested that the more similar the processing demand of the two tasks, the greater their respective performances are correlated and the greater their interaction [41], fundamentally adopting a standard masking argument [42]. The increasing similarity of MOT and VWM task performance with increasingly similar task-processing requirements suggests that interactions observed in the data are likely to be divisible into shared and unshared processes [7]. Our data suggest that scaling the difficulty of each task to the particular subject and removing stimulus-based confounds biases the respective tasks to rely on those shared processes more evenly.

### Working-memory and tracking

One specific model of MOT which intersects partially with the M-WM, the Model of Multiple Identity Tracking, MOMIT [17], proposes the division of labour described above and is worthy of comment. Within this model, the location of each tracked object is stored in visual short-term memory in a temporary store that the authors suggest could be similar to the visuospatial sketchpad of the M-WM. The model also requires a separate temporary store for identity-location bindings, a role that the authors suggest might be fulfilled by the episodic buffer of the M-WM.

There are multiple points at which this process might fail if the required resources are consumed by a concurrent VWM task, even under the assumption that binding is unnecessary during an MOT identity-location task [17]. If we assume that the task of storing the last known location of an MOT target is the responsibility of the visuospatial sketchpad of the M-WM model, then given that the capacity limit of the visuospatial sketchpad is shared between the MOT task and the VWM task, fewer objects can be stored compared to when either task is performed alone. In principle, a strict limit on capacity would distribute the storage between the tasks, effectively halving performance if the slots are distributed evenly, assuming that the constraints of capacity on performance are linear. While there is clear interference between tasks here, the reduction in performance is not 50% so whatever mechanism(s) is/are at play they are able to deal with conflicting demands more than the simplest ‘slot’ model. Indeed, exactly how working memory is limited in capacity is unknown; decay over time, limited resources (or slots), or interference between stored representations are all possibilities although temporal decay seems less likely in light of recent analysis [25].

Another failure could occur when attending to each target in turn to update its current location. If the central executive is unable to efficiently direct attention to each target, it becomes increasingly likely that a distractor will be attended and subsequently tracked instead of the target [8, 24]. This may also account for the decrease in tracking performance observed when individuals were required to simultaneously perform a tone discrimination task [18]; as a purely auditory task, tone discrimination does not involve either visual or spatial processing, and thus would not be expected to place demands on resources shared with an MOT task other than attentional resources, e.g., resources within the central executive.

Mutually disruptive interactions between attention and VWM have also been demonstrated in studies involving an alternative attention-demanding task such as subitising [43], or saccades to peripheral targets [11]. Both of these studies showed an interaction between overt attention and VWM, indicating a more general capacity limit imposed on the common processes underlying memory and attention [25, 29].

### Neurophysiological context

The general consensus in the neuroimaging literature appears to indicate a role for the intraparietal sulcus (IPS) in both visual short-term memory and MOT [44, 45]. Specifically, that the posterior IPS is involved in processing the number of objects held in memory as well as their positions [46–48], but is not necessarily responsible for tracking or for processing the features of objects [44, 45], reminiscent of the shared and non-shared processes outlined above. However, the anterior IPS seems to be involved in processing features of objects, perhaps including velocity information of moving objects. These two regions of the IPS might be the neural correlates of the subcomponent of the visuospatial sketchpad shared by MOT and VWM tasks, as well as the neural correlate of the episodic buffer. The storage of the number and location of objects is a responsibility of the visuospatial sketchpad, while the representation of more complex features of objects, including velocity of moving objects, is a responsibility of the episodic buffer.

### Conclusions

In summary, this study clarifies conflicting data in the literature, providing clear evidence that when an MOT task and a VWM task are performed concurrently, both tasks are disrupted. This might not be the case if either task is not sufficiently challenging to consume the resources available. The pattern of disruption might also be obscured by the order in which tasks are performed, or by the requirement to make a response mid-task. The findings lend support to the conclusion that MOT and VWM tasks share the same resources, and that the shared resources are not limited to those involved in spatial-location processing. These data also place significant constraints on any models of tracking, attention and working memory.

## Supporting information

S1 File Supporting information. Additional data for Experiment 2.
(DOCX)

S1 Fig Mean accuracy in each condition. Single-task and dual-task performance for each observer for the multiple object tracking (MOT) and visual working memory (VWM) tasks. Group analysis to the far right. Error bars represent 95% confidence intervals.
(TIF)
