# Supplementary material for: Shared processing in multiple object tracking and visual working memory in the absence of response order and task order confounds
Source: PLoS One. 2017 Apr 14;12(4):e0175736. doi: 10.1371/journal.pone.0175736 (PMC5391939; doi:10.1371/journal.pone.0175736)
Supplement: S1 File — Additional data for Experiment 2. (DOCX) [file pone.0175736.s001.docx]

## Additional data for Experiment 2

The procedure was the same as Experiment 2 but with reduced calibration time, averaging an hour for each subject for both VWM and MOT combined. The effect of this, essentially having less practiced subjects who may still be improving in performance after initial calibration, is to increase the individual differences as seen in S1 Fig. Overall, however, the group shows a trends toward mutual interaction even in this less than ideal situation. It should be noted that this is still an improvement on the common group-design approach. ­­­­­
