# Supplementary figures and images for: Shared processing in multiple object tracking and visual working memory in the absence of response order and task order confounds
Source: PLoS One. 2017 Apr 14;12(4):e0175736. doi: 10.1371/journal.pone.0175736 (PMC5391939; doi:10.1371/journal.pone.0175736)

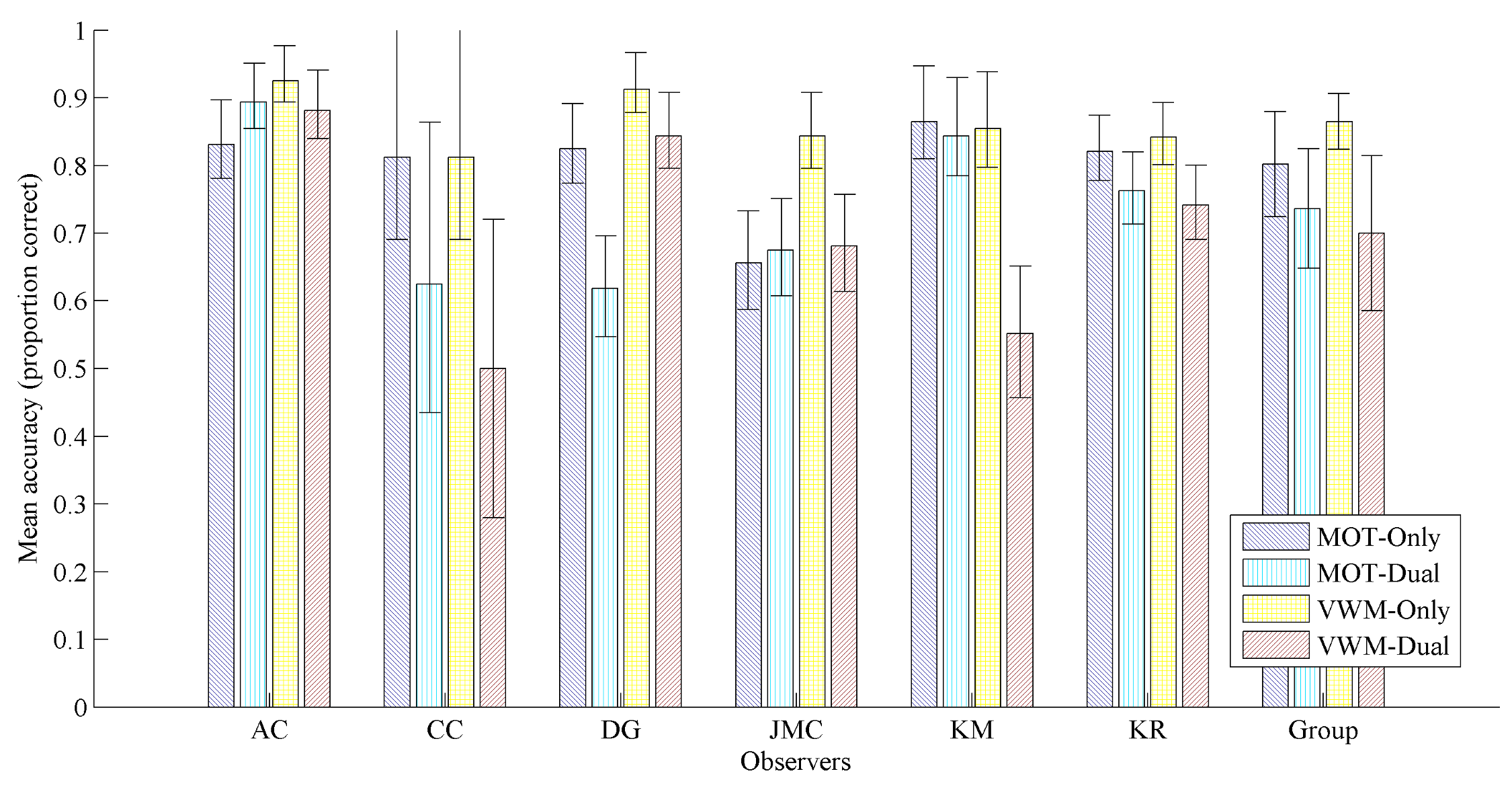

Supplement: S1 Fig — Single-task and dual-task performance for each observer for the multiple object tracking (MOT) and visual working memory (VWM) tasks. Group analysis to the far right. Error bars represent 95% confidence intervals. (TIF) [file pone.0175736.s002.tif]
